# Supplementary figures and images for: Composition of Gut Microbiota in Children with Autism Spectrum Disorder: A Systematic Review and Meta-Analysis
Source: Nutrients. 2020 Mar 17;12(3):792. doi: 10.3390/nu12030792 (PMC7146354; doi:10.3390/nu12030792)

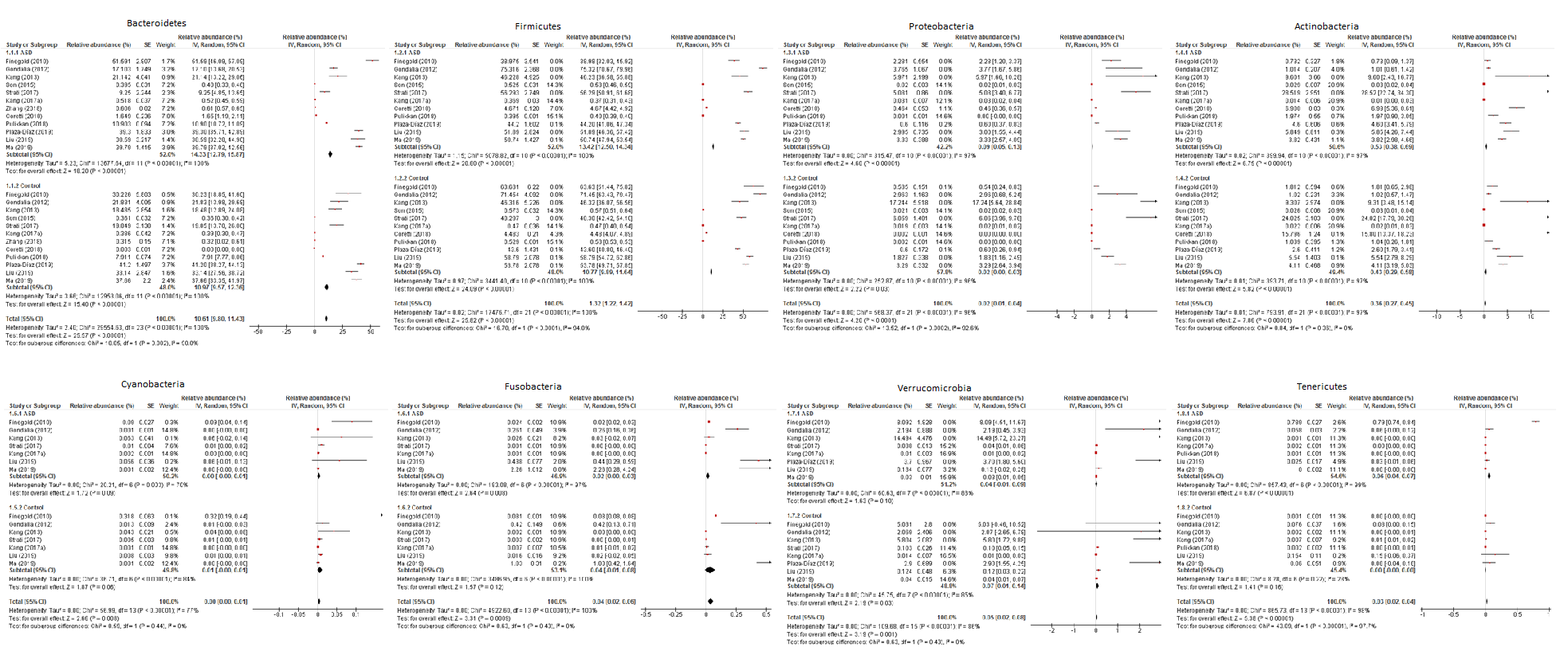

Supplement: Supplementary file 1 [file nutrients-12-00792-s001.zip › Fig S1. Forest plots phyla.png]

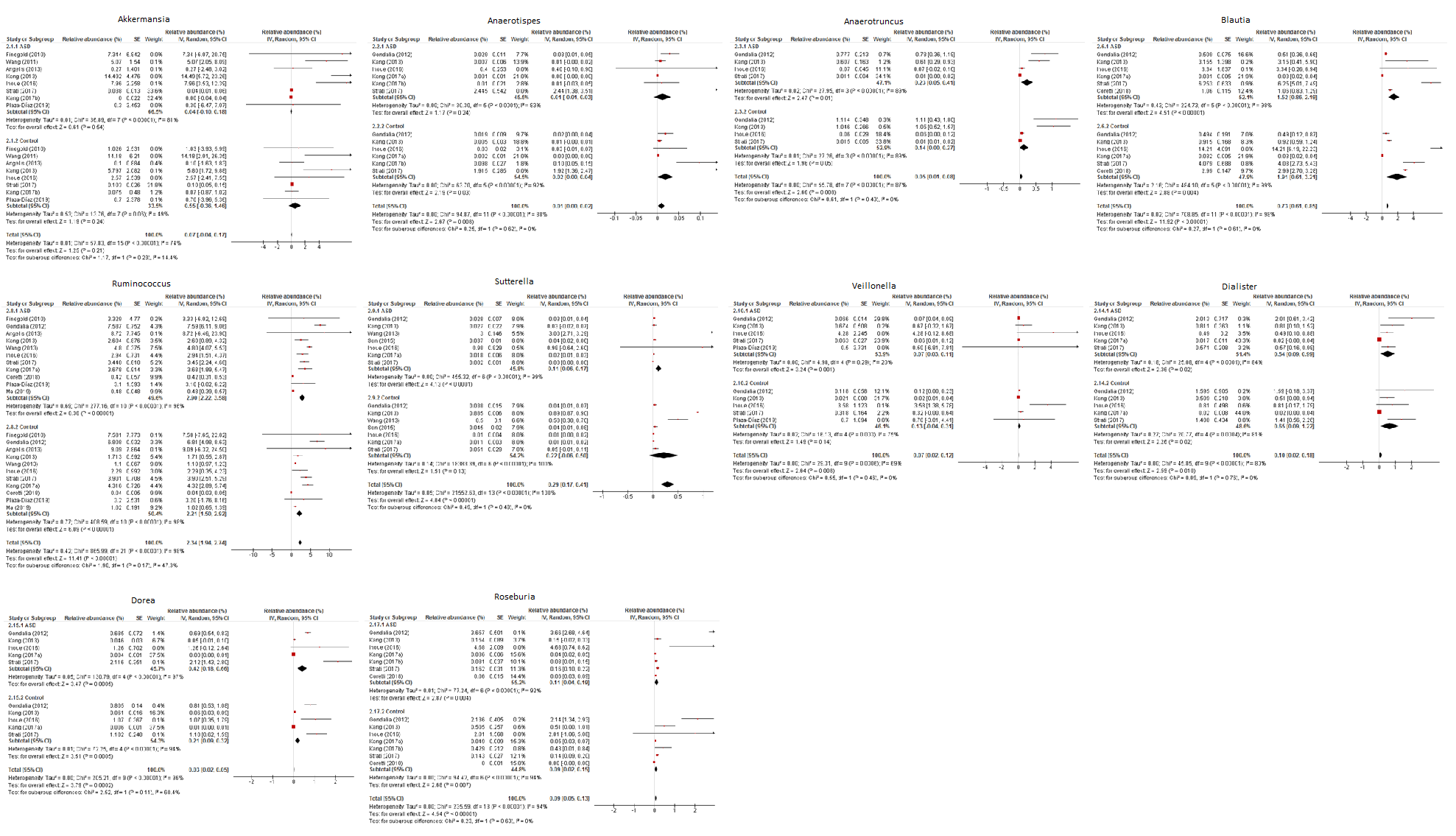

Supplement: Supplementary file 1 [file nutrients-12-00792-s001.zip › Fig S2. Forest plots genera.png]
